# Mechanisms of LncRNA FTX in Regulating Islet Function of Pregnant Mice Born With Low-Protein Diet-Induced Intrauterine Growth Retardation

**DOI:** 10.1007/s43032-025-01870-2

**Published:** 2025-05-08

**Authors:** Li Wang, Yihui Li, Chengting Dai, Yi Yuan, Qingxin Yuan, Jianbo Li

**Affiliations:** 1 Department of Endocrinology and Metabolism, the First Affiliated Hospital with Nanjing Medical University, Nanjing, 210029 China; 2 Department of Endocrinology, The Air Force Hospital From Eastern Theater of PLA, Nanjing, 210008 China; 3 Department of Pathology, Jinling Hospital, Affiliated Hospital of Medical School, Nanjing University, Nanjing, 210016 China; 4 Department of General Medicine, Women’s Hospital of Nanjing Medical University, Nanjing Women and Children’s Healthcare Hospital, Nanjing, 210004 China

**Keywords:** IUGR (intrauterine growth retardation), Pregnancy, LncRNA FTX, Islet function, Diabetes, Low protein diet

## Abstract

Glucose metabolism during pregnancy in adult females born with intrauterine growth restriction (IUGR) remains inadequately understood. This study aims to investigate how LncRNA FTX regulates islet function during pregnancy in F1 female mice born with IUGR (F1 IUGR pregnant mice). A pregnant mouse model was established using F1 female mice born with IUGR (F1 IUGR pregnant mouse model). Intraperitoneal glucose tolerance test (IPGTT), immunohistochemistry (IHC) staining, quantitative real-time PCR (qPCR) were performed in both F1 IUGR and normal mice during pregnancy and non-pregnancy periods. RNA-sequencing was conducted on islets from F1 IUGR and normal pregnant mice. Insulin-related gene expression analysis, cell proliferation, and apoptosis assessment were performed in TC6 cells following FTX knockdown or overexpression. A luciferase reporter assay was conducted to validate the molecular interactions. F1 IUGR pregnant mice exhibited a smaller increase in insulin-staining area and lower upregulation of insulin-related gene expression levels compared to normal pregnant mice. There were 1,007 differentially expressed lncRNAs between F1 IUGR and normal pregnant islets; among these, FTX was down-regulated during pregnancy, although its downregulation in F1 IUGR pregnant mice was less pronounced than in normal pregnant mice. FTX was closely related to cell proliferation activity, apoptosis, insulin-related transcription factor expression. The pten/PI3K/AKT pathway was also regulated by FTX. Luciferase reporter assay confirmed FTX acted as a competing endogenous RNA (CeRNA) to target pten by sponging miR-22-3p. LncRNA FTX regulates islet function during pregnancy in F1 mice born with IUGR via the miR-22-3p/pten axis.

**Graphical Abstract:**

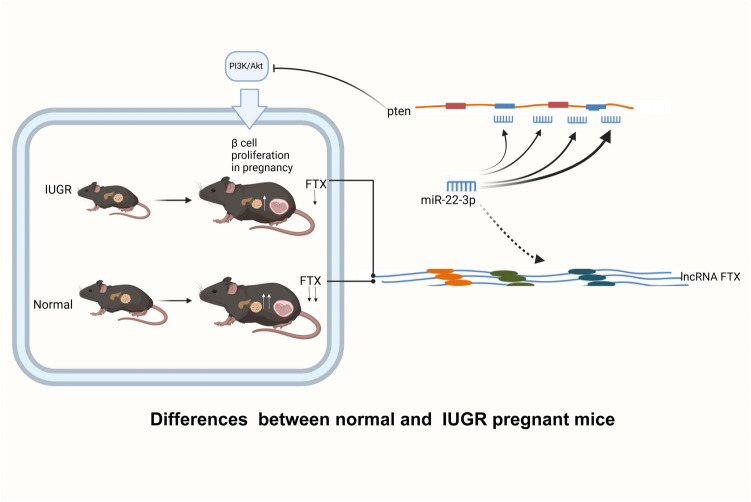

## Introduction

Type 2 diabetes mellitus (T2DM) is a metabolic disease characterized by hyperglycemia, insulin resistance, and relative insulin deficiency, accounting for more than 90% of all diabetes types. The pathogenesis of diabetes is complicated, influenced by both genetic and environmental factors. With the progression of “Developmental Origins of Health and Disease” (DohaD) theory [1], the concept of “fetal-derived diseases” has gained increasing attention. Perinatal environments in utero, intrauterine dysplasia and epigenetic changes will influence the occurrence of metabolic diseases in adulthood, and these individuals are also at a higher risk of experiencing further metabolic abnormalities during pregnancy, leading to a “cross-generational effect” that contributes to the rising incidence of diabetes. Therefore, investigating the pathogenesis of diabetes from the perspective of embryonic development and pregnancy has become a prominent research topic.

Intrauterine growth retardation (IUGR) is a pathological condition that has been widely used to establish experimental animal models for investigating the fetal origins of metabolic diseases such as diabetes [2]. IUGR has been identified as a significant risk factor for type 2 diabetes [3–5]. In newborn IUGR mice, the islet area and serum insulin content were significantly reduced compared to normal mice. While these mice exhibited postnatal catch-up growth, they later became prone to insulin resistance, impaired glucose tolerance, and even diabetes [6]. Additionally, female offspring born with IUGR may experience further disruptions in glucose metabolism and islet dysfunction during pregnancy. However, research on this topic remains limited.

During early pregnancy, maternal insulin sensitivity improves slightly, facilitating glycogen and fat storage to meet the energy demands of later fetal growth. However, as pregnancy progresses, maternal levels of estradiol, progesterone, leptin, cortisol, human placental lactogen (hPL), and placental growth factor (PlGF) undergo significant changes, collectively leading to a 30–70% reduction in insulin sensitivity during mid-to-late pregnancy [7]. This decline limits maternal glucose utilization, ensuring that the fetus receives sufficient glucose and energy for growth and development [8]. Under normal conditions, maternal pancreatic β-cells compensate by proliferating and reducing apoptosis to maintain glucose homeostasis. However, when females born with IUGR, whose β-cell development and islet function were compromised early in life, face the additional metabolic demands of pregnancy, their adaptive responses and underlying mechanisms poorly understood.

Environmental factors early in life, such as maternal nutritional deficiencies, alcohol exposure, hypoxia, and drug abuse, can induce epigenetic modifications with lasting effects on offspring. Epigenetic regulation refers to heritable changes in gene expression that occur independently of DNA sequence alterations, including DNA methylation, histone modification, chromatin remodeling, and non-coding RNA activity. Long non-coding RNA (LncRNA), a class of non-coding RNAs longer than 200nt, have recently emerged as key regulators in islet cell development, T2DM and related complications, and the pathogenesis of IUGR. Our preliminary study revealed that lncRNA Tug1 plays a role in IUGR-associated islet dysfunction in T2DM [6]. Additionally, lncRNA plays a role in pregnancy and its complications. LncRNA Gm16308 (Lnc03) regulated by prolactin could be a positive regulator of β-cell proliferation during pregnancy [9]. LncRNA five Prime to Xist (FTX), located at the X chromosome inactivation (XCI) center upstream of the Xist gene, has been extensively studied in tumorigenesis and cancer progression. FTX can also function as an endogenous competitive RNA (CeRNA) by interacting with miRNAs, thereby indirectly regulating the downstream expression of miRNA targets, such as mRNA. For instance, FTX affected osteosarcoma cell proliferation and migration through the miR- 320 A/TXNRD1 axis [10]. FTX might also play a role in glucose metabolism. Guo [11] et al. found serum FTX levels were elevated in patients with T2DM, consistent with Huang [12] et al.'s conclusion that FTX was upregulated in peripheral blood monocytes of individuals with T2DM. However, its expression and function in pancreatic activity during pregnancy remain unclear.

In this study, we established an IUGR pregnancy model using F1 female mice born with IUGR. High-throughput sequencing identified differentially expressed lncRNAs in the islets of normal and F1 IUGR pregnant mice, with lncRNA FTX selected for further investigation due to its potential role in regulating islet function during pregnancy. We hypothesize that FTX expression is upregulated in islets of F1 IUGR pregnancy compared to normal pregnancy and that it regulates microRNA- 22 - 3p activity through a sponge effect, thereby influencing the expression of its target mRNA, pten (a negative regulator of the PI3 K/AKT pathway), ultimately modulating β-cell function, proliferation, and apoptosis during pregnancy.

Elucidating the regulatory role and underlying mechanism of lncRNA FTX in islet function during IUGR pregnancy could provide valuable insights into the developmental origins of T2DM. Furthermore, these findings may offer new perspectives for improving adverse intrauterine environments and advancing early prevention and treatment strategies for diabetes.

## Materials and Methods

### Model of Normal and IUGR Pregnant Mice

**Fig. 1:**
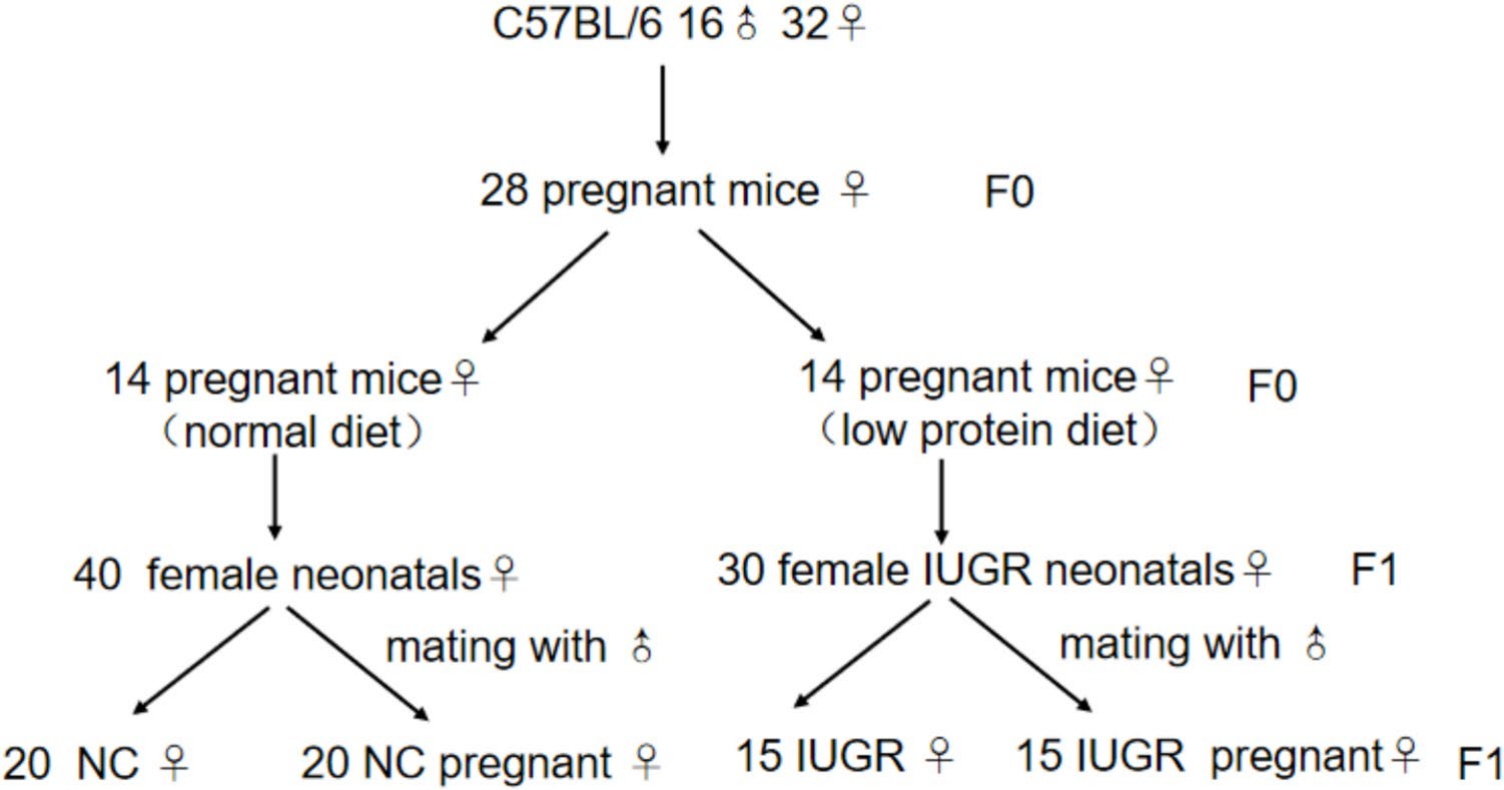
Model of normal and IUGR pregnant mice

32 female and 16 male C57BL/6 mice aged 12 weeks were provided by the Animal Center of Nanjing Medical University, and raised in Emei Animal House of Nanjing Medical University. Animal experiments was approved by the Animal Protection and Ethics Committee of Nanjing Medical University (Approval No. IACUC- 1703003). After 1 week of acclimatization, female mice were paired with males at the ratio of 2:1 overnight. In the next morning, sperm embolus were found in the vaginal and sperm were confirmed in vaginal smears for the verification of pregnancy. 28 pregnant mice (F0 generation) were divided into two groups. 14 normal pregnant mice (F0) were given standard reproductive diet (20% protein, 1558 kJ/100 g) from the beginning of pregnancy, while the other 14 (F0) were fed a low-protein diet (purchased from Beijing XieTong Biology limited company, 8% protein, 1558 kJ/100 g) until weaning. As a result, 50 symmetrical IUGR neonatal mice (F1 generation) were obtained, defined as having a birth weight below the 10 th percentile for the same gestational age. These mice were selected from a total of 98 neonates in the low-protein diet group. Then, 15 out of 30 female F1 IUGR mice aged 12 weeks were paired with age-matched control males to obtain F1 IUGR pregnant mice, and were fed a standard reproductive diet from conception. For clarity, throughout the manuscript, “IUGR pregnant mice” specifically refers to F1 females exposed to a low-protein diet in utero that later became pregnant, while “IUGR” refers to non-pregnant F1 IUGR mice born from F0 dams fed a low-protein diet. The process of a single modeling procedure is illustrated in Fig. 1.

### Intraperitoneal Glucose Tolerance Test (IPGTT) and Serum Insulin Level

An intraperitoneal glucose tolerance test was performed by administrating 2 g/kg of glucose to 12 h fasted mice at two different time points: (1) at 4 weeks of age in postnatal F1 IUGR mice to assess the long-term metabolic effects of intrauterine growth restriction (in utero IUGR effect), and (2) on the 12 th day (similar to the glucose tolerance test performed in pregnant women at 24 weeks of gestation; the normal gestation period for mice is 18–21 days) of pregnancy in F1 IUGR pregnant mice to evaluate glucose metabolism during pregnancy. Blood samples (50ul) for insulin measurement were collected from the tail vein at 0, 15, 30, 60, and 90 min after glucose injection from four groups: normal control (NC), normal pregnant mice (NC pregnancy), F1 IUGR mice (IUGR), and F1 IUGR pregnant mice (IUGR pregnancy). The blood glucose and serum insulin level were measured using a glucometer (Roche Corporation, America) and a mouse insulin ElISA kit (10–1247 - 01, Mercodia, Sweden) respectively.

### Immunochemistry (IHC)

At the 15 th day of pregnancy, three 14-week-old mice from each group— NC, NC pregnancy, IUGR, and IUGR pregnancy—were deeply anesthetized with 1% pentobarbital sodium. After the eyeballs were removed and blood was let out to ensure a clear surgical field, the abdomen was opened, and the pancreas was carefully extracted. Fetuses were carefully removed and disposed of in accordance with institutional ethical guidelines. Isolated pancreas were immersion-fixed in 4% paraformaldehyde, routinely processed, embedded in paraffin, and sectioned. Tissue sections were dewaxed and rehydrated, and subjected to antigen retrieval with citrate buffer (pH 6.0) in a pressure cooker for 3 min. Then, the sections were incubated in 3% H_2_O_2_ for 30 min to inactivate endogenous peroxidase. Subsequently, they were immunostained with rabbit anti-insulin antibody (1:250, sc- 9168, Santa Cruz) overnight at 4℃ and incubated in secondary antibody (1:500, HRP Conjugated Goat anti-Rabbit IgG h + 1 antibody, abs20040 ss) at room temperature for 2 h. The labeled antigen was visualized by 3,3′-Diaminobenzidine tetrahydrochloride (DAB) and finally counter-stained with hematoxylin. IHC-stained pancreatic tissue sections were imaged with a microscope (ZEISS, Axioscope 5/7). Images were opened in ImageJ and converted to 8-bit grayscale, and the optimal DAB channel (typically green) was selected via Split Channels. After threshold adjustment (Image → Adjust → Threshold), the positive area was measured (“Analyze → Measure” with “Limit to Threshold” enabled). The total tissue area was defined manually, and the positive staining percentage was calculated as: Positive Staining (%) = Positive Staining Area/Total Tissue Area × 100%. Finally, data were exported to GraphPad Prism for statistical analysis.

### Extraction of Primary Mouse Islet Cells

After being deeply anesthetized with 1% pentobarbital sodium, nine 14-week-old mice from four experimental groups were sacrificed by enucleation and exsanguination. The common bile duct was isolated and punctured, followed by the injection of cold collagenase for tissue digestion. The distended pancreas was removed and placed in pre-cooled 15 mL centrifuge tubes containing 2–3 mL collagenase. Tubes were placed in a 37℃ water bath for 7–9 min for static digestion. To halt the digestion process, 4–6 mL HBSS containing 10% serum was added to each tube. The digested mixture was then passed through a cell sieve to isolate the islets, which were further purified with Histopaque 1077. The islets were counted under a microscope and used for the subsequent RNA-Sequencing and qRT-PCR experiment.

### RNA-Sequencing and Analysis

Total RNA was extracted from islet cells isolated from three NC pregnancy mice and three F1 IUGR pregnant mice, and RNA sequencing was performed by Guangzhou Gidio Biotechnology Limited Company. RNA concentration and purity were assessed using a NanoDrop microspectrophotometer. After the removal of ribosomal RNA, both coding and non-coding RNAs were retained to maximize yield. The RNA was then fragmented into short, random segments. The first strand of complementary DNA (cDNA) was synthesized using random hexamers as primers and the fragmented RNA as a template. Following this, deoxynucleoside triphosphates (dNTPs) (with dUTP substituting for dTTP), RNase H, DNA polymerase I, and a buffer solution were added for the synthesis of the second strand of cDNA. The second strand was subsequently degraded by uracil-N-glycosylase (UNG). The resulting fragments were selected for PCR amplification through agarose gel electrophoresis. Finally, the library was sequenced using the Illumina HiSeq™ 4000 platform.

After obtaining clean data by filtering the raw data, the reads were aligned to the mouse reference genome (Ensembl version 104) using Tophat2 (2.1.1). To identify known and noval transcripts, the transcripts were reconstructed with Cufflinks. The coding ability of new transcripts was predicted using CPC, CNCI software, thus the newly predicted lncRNAs were obtained. The mRNA and lncRNA expression levels in the samples were analyzed respectively, and the differential expression between groups were assessed using the edgeR package. |log2 FC|> 1, *p* < *0.05* indicates a difference.

The target mRNA of lncRNA was predicted by Pearson correlation coefficient and RNAplex. The Gene Ontology (GO) function annotation analysis and pathway enrichment analysis in the Kyoto Encyclopedia of Genes and Genomes (KEGG) database were performed for the target genes.

### Cell Culture and Transfection

Mouse β-cell line TC6 were obtained from the Endocrinology laboratory of the first Affiliated Hospital of Nanjing Medical University. The components of the medium were DMEM medium (Invitrogen, USA), 10% FBS, 1% double antibody (100U/mL penicillin and 100 μg/mL streptomycin). The cells were cultured in a 5% CO2, 37 °C incubator, with media changed daily. Passage is performed when the cell density reaches approximately 80%. To silence the lncRNA expression, the specific siRNA targeting FTX were synthesized by GenePharma (Shanghai, China). TC6 was transiently transfected with si-FTX, si-NC and lipofectamine 3000 (Invitrogen, USA). The plasmid for FTX was synthesized by Obio Technology (Shanghai, China) to enhance the FTX expression. TC6 was transiently transfected with pcDNA-empty, pcDNA-FTX, P3000 (Invitrogen, USA) and lipofectamine 3000. Each experimental group had three duplicate holes.

### Luciferase Reporter Assays

The wild-type (WT) and mutant (MUT) 3′-untranslated region (UTR) binding site were inserted into the pmirGLO dual-luciferase vector (Obio Technology, Shanghai, China) to generate FTX WT/MUT and pten WT/MUT plasmids. Then, the constructed luciferase reporter plasmids were co-transfected with or without the miRNA- 22 - 3p mimics into TC6 cells. After 48 h of transfection, luciferase activity was measured by a dual luciferase assay system (Cat#E1910, Promega, USA) on a HT microplate reader (BioTek). Each experimental group had three duplicate holes.

### Cell Vability Assay

A Cell Counting Kit 8 (CCK- 8, Cat#HY-K0301, MCE, USA) was used to measure the cell activity of the transfected cells according to manufacturer’s instructions. The transfected cells were seeded into a 96-well plate (10^3^- 10^4^ cells per well), and CCK- 8 reagent (10 µL) was added into each well for another 2 h incubation. The absorbance was measured at 450 nm wavelength with a microplate reader (BioTek). Each experimental group had three duplicate holes.

### Apoptosis Assay

The cells were plated in a 6-well plate at a density of 1 × 10^5^ cells/well. Annexin V-FITC/PI apoptosis detection kit (Cat#KGA108, KeyGen Biotech, China) was used to test cell apoptosis after transfection. After digested by pancreatin and washed with PBS, resuspended cells were stained with 5μL Annexin V-FITC and 5μL Propidium Iodide (PI) in the dark for 15 min respectively. The flow cytometry (BD Biosciences, China) was performed to detect the cells stained with AV and PI. The standard gating process for analyzing cell apoptosis using AV/PI double staining in FlowJo is as follows. FCS files were loaded into FlowJo (version 10). Forward scatter (FSC) and side scatter (SSC) were used to define the main cell population and exclude cellular debris and non-cellular events by setting a P1 gate. A density plot was generated by plotting Annexin V (X-axis) vs. PI (Y-axis) within the selected P1 population. A quadrant gate (Q1–Q4) was manually defined to classify cell populations. The proportion of apoptotic cells was determined by summing the percentages of Q2 (late apoptotic) and Q3 (early apoptotic) populations. These values were obtained from the Statistics panel in FlowJo and used for subsequent data analysis. Each experimental group had three duplicate holes.

### Quantitative Real-Time Polymerase Chain Reaction (qRT-PCR)

Total RNA was extracted from tissues or cells using trizol reagent (Invitrogen Life Technologies, USA) and converted into cDNA by PrimeScript™ Reverse Transcription reagent kits (Cat#RR036 A,Takara, Japan). All cDNA samples were mixed with SYBR Green PCR Master Mix (Cat#Q141,Vazyme, China) for PCR amplification on Step-One Plus System (Applied Biosystems, USA). RNA level was calculated by 2^−△△Ct^ method and normalized to β-actin. All the primers are listed in Table 1.

**Table 1:** The primer sequences of genes

gene	F Primer	R Primer
β-Actin	GCTACAGCTTCACCACCACAG	GGTCTTTACGGATGTCAACGTC
FTX	AAGATCTCCGCTGCCAGATG	CTGCTCCTGTGCCACGAATA
Insulin 1	CAATCATAGACCATCAGCAAG	AGAAACCACGTTCCCCAC
Insulin 2	CCCAGGCTTTTGTCAAACAG	GTGCCAAGGTCTGAAGGTC
NeuroD	CAAAGCCACCGGATCAATCTT	TACGCACAGTGGATTCGTTT
Pten	GAAAGGGACGGACTGGTGTA	AGTGCCACGGGTCTGTAATC
Pdx- 1	AATTCTTGAGGGCACGAGAG	GCCCAGGTTGTCTAAATTGG
Glut2	ATCGCCCTCTGCTTCCAGTAC	GAACACGTAAGGCCCAAGGA
MiR- 22 - 3p	AAGCTGCCAGTTGAAGAACTGTA	GCTGTCAACGATACGCTACGTAAC

### Western Blotting

Total protein was extracted from TC6 cells using Ripa lysate. BCA Protein Assay Kit (Beyotime, China) was used to detect the concentration of total protein. Then, the protein samples were electrophoretically separated by 10% SDS-PAGE gel (Cat#PG112, Epizyme, China) and transferred to PVDF membrane. The PVDF membrane was sealed in the rapid sealing solution for half an hour and incubated in primary antibody (mouse anti-tublin sc5286 1:500, caspase- 3 sc- 56053 1:500, bcl- 2 sc- 23960 1:500, bax sc- 7480 1:500 purchased from SANTA CRUZ, rabbit anti-PI3 K DF2626 1:1000 purchased from Affinity, rabbit anti-pAKT T40067 F 1:1000, AKT T55561 F 1:1000, p-PI3 K T40116 F 1:1000 purchased from Abmart, mouse anti-β actin 8H10D10 1:1000 purchased from Cell Signaling) diluent at 4 °C with shaking overnight. Next morning, the membrane was incubated in secondary antibody (peroxidase-conjugated goat anti-mouse/rabbit IgG H + L, 1:10000, ZSGB-BIO) at room temperature for 2 h. Finally, TBST was cleaned for 3 times before exposure. Tublin or β-actin were used as the internal reference, and the relative expression levels of protein were quantitatively analyzed by ImageJ software.

## Statistical Analysis

Each experiment in this study was independently performed three times. SPSS25.0 and Graphpad prism 8.4.3 software was used for statistics in this experiment. Comparisons among the four groups — NC, NC pregnancy, IUGR, and IUGR pregnancy—were performed using two-way analysis of variance (ANOVA). The experimental data were expressed as individual data points and the mean ± SD. For comparisons among multiple independent groups, one-way ANOVA was used; for comparisons between two independent groups, the independent t-test was employed. *p < 0.05, **p < 0.01, ***p < 0.001, ****p < 0.0001 indicated statistically significant differences.

## Results

### Islet Proliferation Occurred During Pregnancy, but Was Weaker in F1 IUGR Pregnant Mice

**Fig. 2:**
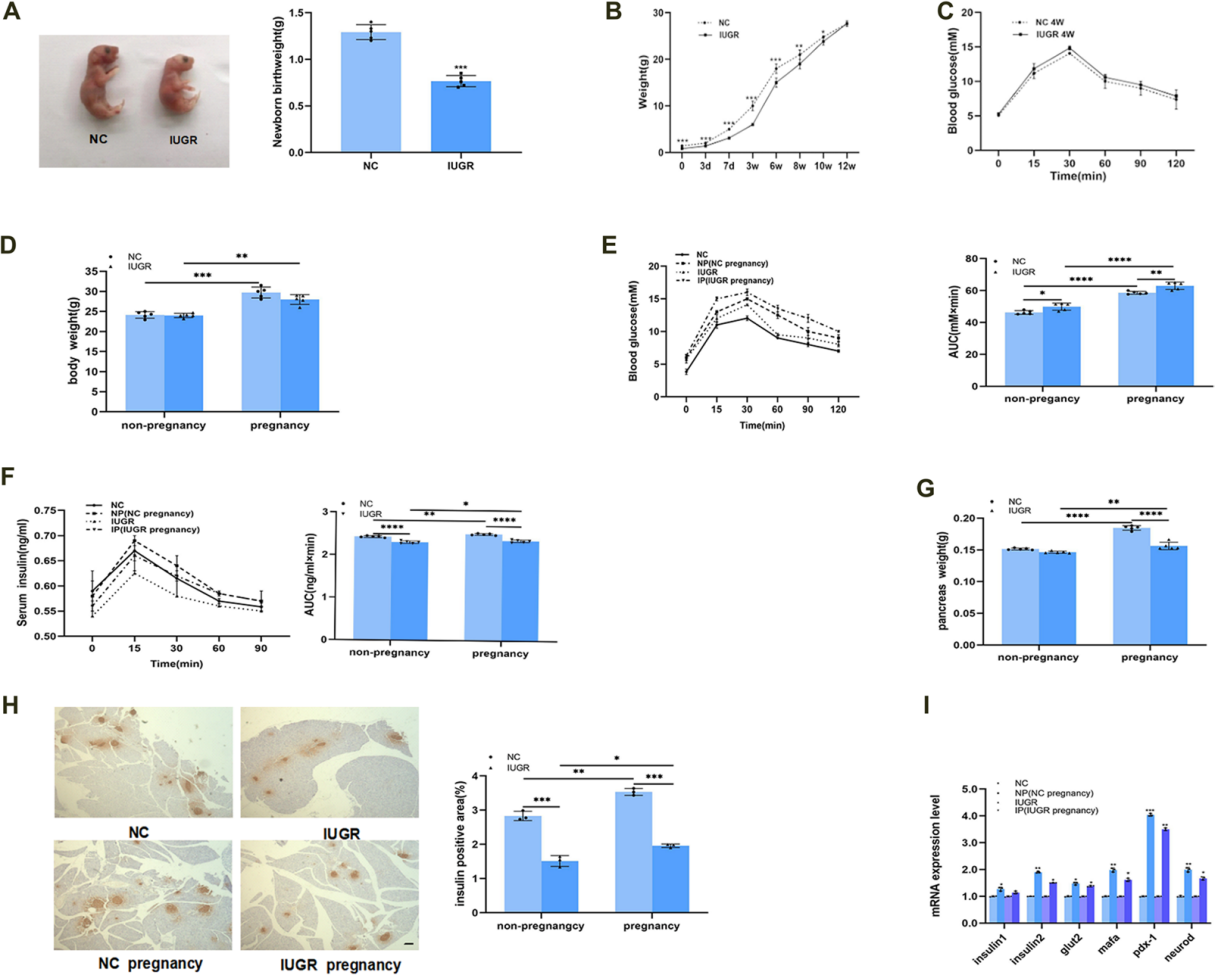
Islet proliferation occurred during pregnancy, but weaker in IUGR mice. (**A**) The appearance and birthweight of normal control and IUGR mice. (**B**) The weight of normal control and IUGR mice during the growth process. (**C**) Intraperitoneal glucose tolerance test of normal control and IUGR mice at 4 weeks. (**D**) The weight of NC, NC pregnancy, IUGR, IUGR pregnancy (gestational day12). (**E**) Blood glucose and (**F**) serum insulin levels of NC, NC pregnancy, IUGR, IUGR pregnancy (gestational day12). (**G**) Pancreas weight of NC, NC pregnancy, IUGR, IUGR pregnancy (gestational day15). (**H**) Pancreatic immunohistochemistry for insulin of NC, NC pregnancy, IUGR, IUGR pregnancy (gestational day15), Scale-bar = 50 µm. (**I**) Expression levels of genes associated with insulin synthesis and secretion of NC, NC pregnancy, IUGR, IUGR pregnancy (gestational day15), compaired with non-pregnant. NC, Normal Control mice; NP, Normal Control pregnant mice; IUGR, Intrauterine Growth Restriction; IP, Intrauterine Growth Restriction pregnant mice. *p < 0.05, **p < 0.01, ***p < 0.001, ****p < 0.0001

Mothers (F0) fed a low protein (8%) diet had low-birthweight babies than those fed a standard reproductive diet. The appearance of symmetrical F1 IUGR mice at birth was significantly smaller than that of normal newborn mice, indicating successful modeling of IUGR mice (Fig. 2A). The maternal low-protein diet was maintained until postnatal day 21 (weaning). Body weight of F1 offspring was monitored from birth to 12 weeks of age. F1 IUGR mice displayed catch-up growth after weaning, and their body weight matched that of normal controls by week 10 (Fig. 2B). IPGTT performed at 4 weeks of age revealed no significant differences between postnatal F1 IUGR mice and age-matched controls (Fig. 2C). By gestational day 12, body weight had significantly increased in both normal control and F1 IUGR pregnant mice (Fig. 2D). Blood glucose (Fig. 2E) and insulin levels (Fig. 2F) increased during pregnancy in both groups. However, F1 IUGR mice exhibited higher blood glucose levels and lower insulin levels than normal mice, both in the non-pregnant state and during pregnancy. At gestational day 15, mice from four experimental groups were sacrificed. Pancreatic weight significantly increased during pregnancy (Fig. 2G); however, the increase was significantly lower in F1 IUGR pregnant mice than in normal pregnant mice. IHC analysis revealed that the insulin-staining area in F1 IUGR mice was smaller than that in normal mice, but both groups exhibited an increase during pregnancy. Nevertheless, the insulin-staining area in F1 IUGR pregnant mice remained significantly smaller than that of normal pregnant mice (Fig. 2H). The expression of genes involved in insulin synthesis and secretion was assessed by qRT-PCR. These genes were upregulated during pregnancy in both normal and F1 IUGR pregnant mice (Fig. 2I); however, the magnitude of upregulation was lower in F1 IUGR pregnant mice, indicating impaired islet function in this group.

### Differentially Expressed lncRNAs in Islets of F1 IUGR Pregnant Mice and Potential Functional Relevance of lncRNA FTX

**Fig. 3:**
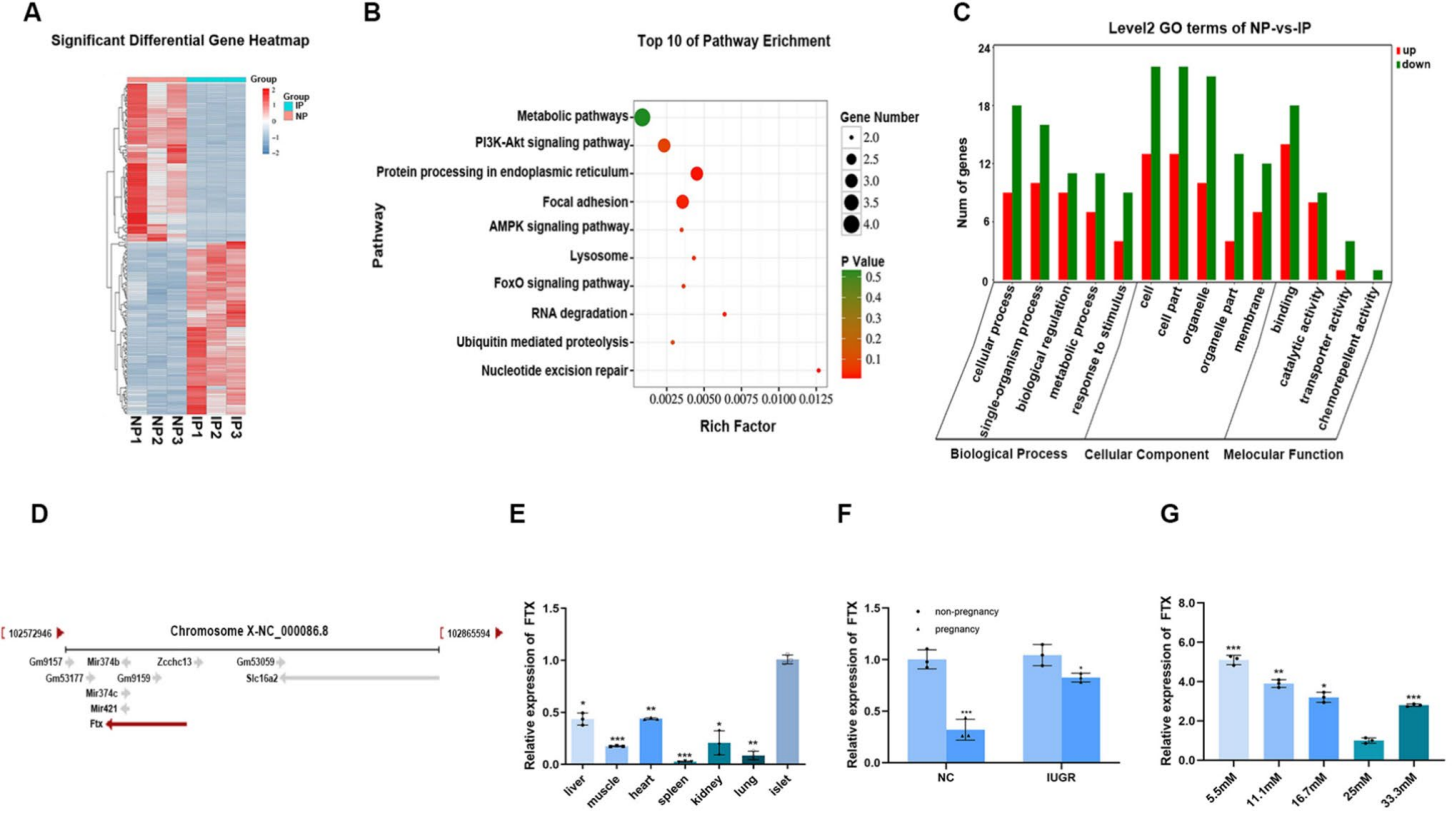
LncRNA FTX was insufficiently down-regulated in IUGR pregnant mice. The (**A**) heatmap of differentially expressed lncRNAs between IUGR pregnant mice and normal pregnant mice. The (**B**) KEGG enrichment and (**C**) GO analysis on target mRNA of differentially expressed LncRNA. (**D**) The localization of lncRNA FTX in chromosome X. (**E**) Expression levels of lncRNA FTX in different tissues of 8-week-old normal control mice, compared with islets. (**F**) LncRNA FTX expression level in islets of NC, NC pregnancy, IUGR, IUGR pregnancy, compared with non-pregnancy. (**G**) The expression level of LncRNA FTX in TC6 cells under different glucose concentrations, compared with 25 mM. NC, Normal Control mice; NP, Normal Control pregnant mice; IUGR, Intrauterine Growth Restriction; IP, Intrauterine Growth Restriction pregnant mice. *p < 0.05, **p < 0.01, ***p < 0.001

Total RNA from the islets of normal pregnant (NC pregnancy) and F1 IUGR pregnant mice was sent to Gene Denovo Biotechnology Co., LTD for sequencing. The analysis identified 1,007 differentially expressed lncRNAs between the two groups, with 483 up-regulated and 524 down-regulated in F1 IUGR pregnant mice compared to normal pregnant mice (Fig. 3A). KEGG enrichment (Fig. 3B) and GO analysis (Fig. 3C) on target mRNAs of these differentially expressed lncRNAs indicated associations with metabolic pathways, PI3 K/AKT signaling pathway, AMPK signaling pathway, etc. Based on the sequencing analysis, lncRNAs exhibiting significant expression differences and potential involvement in islet biology were selected for further validation, including Meg3, Dleu2, and FTX (Table 2). Among them, FTX showed the highest expression level in islets and greatest expression differences between normal and F1 IUGR pregnant mice. Then, we focused on lncRNA FTX. LncRNA five Prime to Xist (FTX) is expressed in both humans and mice, located in the X chromosome inactivation center (XCI) and upstream of Xist gene (Fig. 3D, cited from National Center For Biotechnology Information). We detected the expression level of lncRNA FTX in liver, heart, spleen, lung, kidney, muscle tissue and islet of 8-week-old normal control mice, observing notably higher expression in islet tissue (Fig. 3E). We also assessed FTX expression in islets from each experimental group, revealing that FTX was downregulated during pregnancy in both normal and F1 IUGR pregnant mice compared to their non-pregnant counterparts, though the extent of downregulation was less pronounced in F1 IUGR pregnant mice (Fig. 3F). As insulin is regulated by glucose concentration, we further investigated whether FTX expression is glucose-responsive. TC6 cells were stimulated by different glucose concentrations (5.5 mM, 11.1 mM, 16.7 mM, 25 mM, 33.3 mM), of which 25 mM was the glucose concentration in TC6 cell culture medium. Interestingly, FTX expression was up-regulated in low-glucose (5.5 nM, 11.1 mM, 16.7 nM) and high-glucose (33.3 mM) medium relative to 25 mM glucose concentration (Fig. 3G), indicating that glucose regulates FTX expression.

**Table 2:** Three differentially expressed lncRNAs between IUGR pregnant mice and normal pregnant mice

ID	LncRNA	Log_**2**_**(**FC**)**	P Value	Up or down regulated
ENSMUST00000124106	Meg3 - 201	9.966	0.002	up
ENSMUST00000180917	Dleu2 - 202	1.607	0.004	up
ENSMUST00000236107	FTX- 207	7.492	0.001	up

### Down-Regulation of LncRNA FTX Promoted Cell Activity and the Expression of Genes Related to Insulin Synthesis and Secretion, and Inhibited Cell Apoptosis

**Fig. 4:**
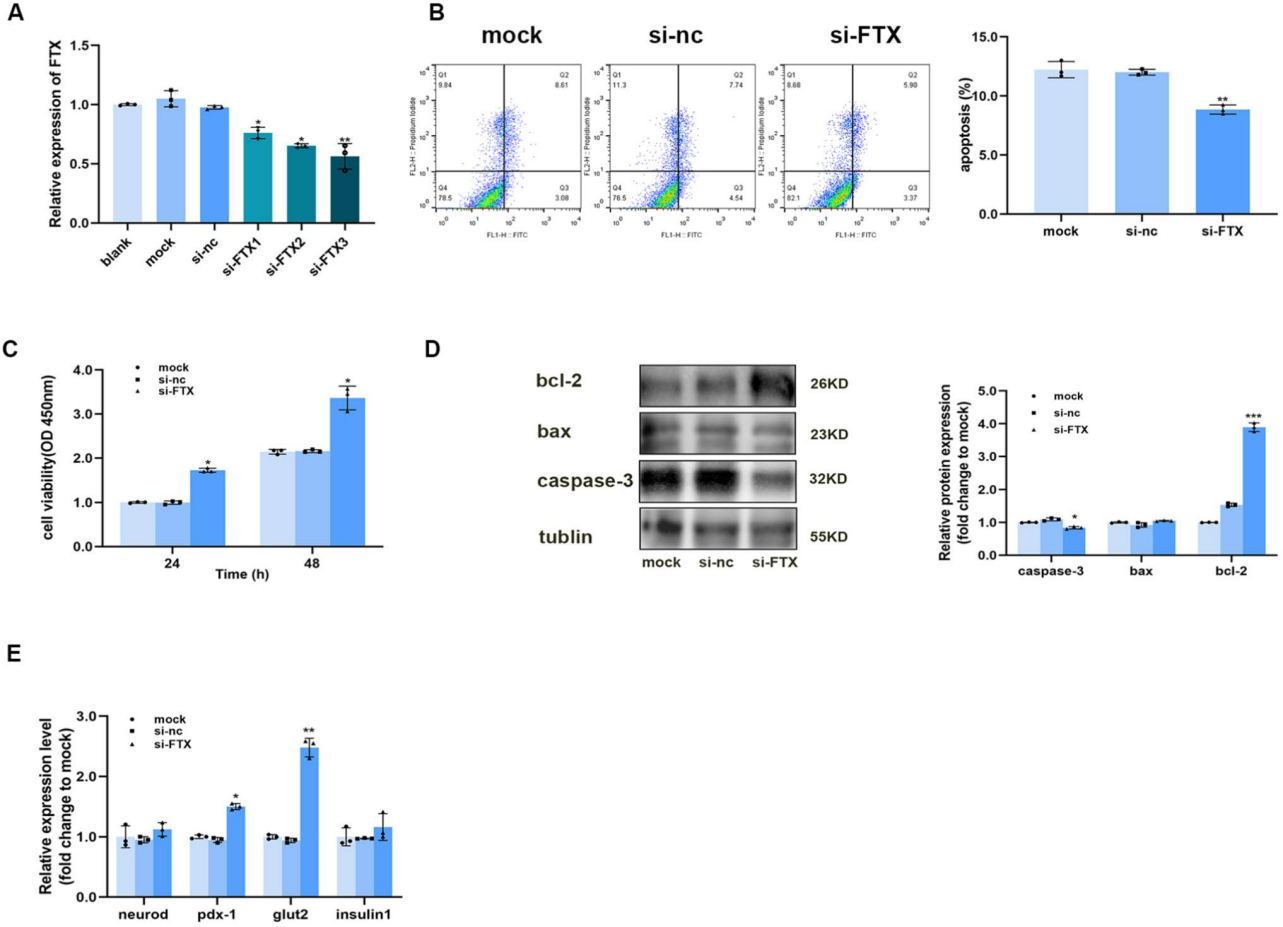
Down-regulation of LncRNA FTX promoted cell proliferation and the expression of genes related to insulin synthesis and secretion, and inhibited cell apoptosis. (**A**) Interference efficiency of three LncRNA FTX interfering sequences in TC6 cells, compared with the control group. (**B**) The representative graph of apoptosis ratio of TC6 cells after downregulation of lncRNA FTX, compared with the mock group. (**C**) Proliferation activity of TC6 cells after FTX intervention 24 h and 48 h later, compared with the mock group. (**D**) Expression of apoptosis-related proteins in TC6 cells after downregulation of lncRNA FTX, compared with the mock group. The expression of insulin-related genes after lncRNA FTX was down-regulated in TC6 cells at (**E**) nucleic acid level, compared with the mock group. *p < 0.05, **p < 0.01, ***p < 0.001

In order to investigate the effect of lncRNA FTX on mouse islet cell line β-TC6, we ordered three interference sequences of lncRNA FTX from Shanghai GenePharma Co., LTD. The interference efficiency was detected by qRT-PCR, which indicated that si-FTX3 had the best interference efficiency (Fig. 4A). Therefore, si-FTX3 was selected for subsequent experiments. Our previous study demonstrated that FTX expression was down-regulated (Fig. 3F) and that the insulin-staining area increased during pregnancy (Fig. 2H), leading us to hypothesize that lncRNA FTX may be involved in maintaining the population of islet β-cells. Flow cytometry revealed a significant reduction in apoptosis in the si-FTX group (Fig. 4B). Additionally, CCK- 8 assays indicated that FTX downregulation significantly enhanced TC6 cell viability at 24 and 48 h post-transfection (Fig. 4C). Western blot analysis of apoptosis-related proteins in TC6 cells following FTX knockdown showed a marked decrease in pro-apoptotic proteins Bax and Caspase- 3, alongside an increase in the anti-apoptotic protein Bcl- 2 (Fig. 4D). The synthesis and secretion of insulin require the participation of transcription factors. QRT-PCR revealed that the expression of insulin, neurod, pdx- 1 and glut2 were increased after lncRNA FTX was knocked down in TC6 cells (Fig. 4E). These results suggested that down-regulation of LncRNA FTX promoted cell activity and the expression of genes related to insulin synthesis and secretion, and inhibited cell apoptosis.

### Overexpression of lncRNA FTX Inhibited Cell Activity and the Expression of Insulin-Related Genes, and Promoted Apoptosis

**Fig. 5:**
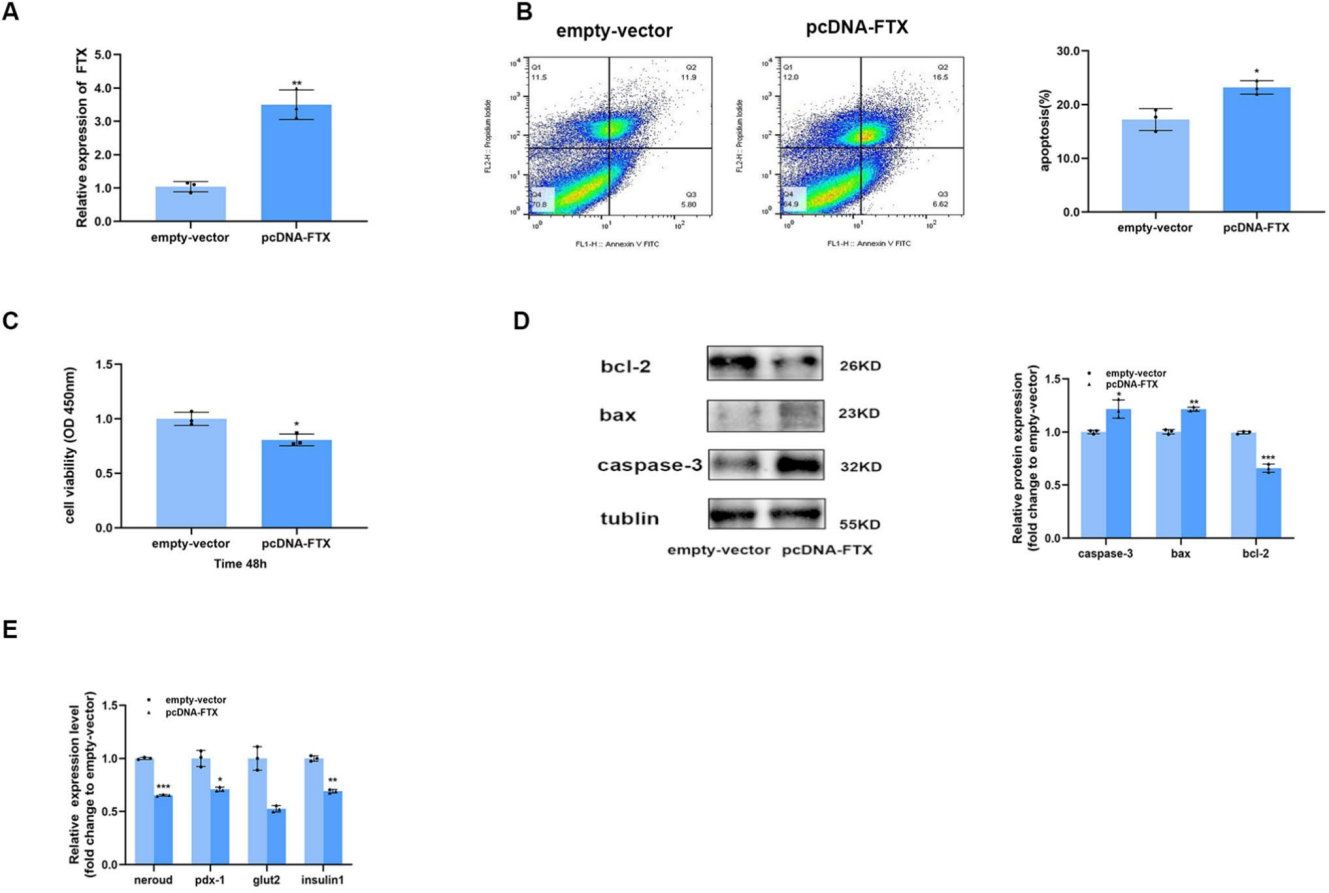
Overexpression of lncRNA FTX inhibited cell proliferation and the expression of insulin-related genes, and promoted apoptosis. (**A**) The overexpression efficiency of pcDNA-FTX in TC6 cells, compared with the empty-vector group. (**B**) The representative graph of apoptosis ratio of TC6 cells after the overexpression of lncRNA FTX, compared with the empty-vecor group. (**C**) The proliferation activity of TC6 cells after FTX was over-expressed, compared with the empty-vecor group. (**D**) The expression of apoptosis-related protein after the overexpression of lncRNA FTX in TC6 cells, compared with the empty-vecor group. The expression of insulin-related genes at (**E**) nucleic acid level after the overexpression of FTX in TC6 cells, compared with the empty- vector group. *p < 0.05, **p < 0.01, ***p < 0.001

To assess the effects of lncRNA FTX overexpression in TC6 cells, we used a PcDNA-FTX construct provided by Shanghai Obio Biotechnology Co. Initially, we confirmed the overexpression efficiency in TC6 cells, observing a significant increase in lncRNA FTX expression after PcDNA-FTX transfection (Fig. 5A). Flow cytometry suggested that the apoptosis ratio of TC6 cells in the overexpression group was higher than that of the empty-vector group (Fig. 5B). Moreover, CCK8 implied that the activity of TC6 cells in the overexpression group was worse than that of empty-vector group (Fig. 5C). WB showed that the expression levels of Caspase- 3 and Bax increased and Bcl- 2 decreased in the overexpression group (Fig. 5D). The mRNA expression level of insulin1, neurod, pdx- 1 and glut2 all displayed a downward trend (Fig. 5E). These results implied that overexpression of lncRNA FTX inhibited cell activity and the expression of insulin-related genes, and promoted apoptosis.

### FTX Was a Sponge of miR- 22 - 3p Which Regulated Pten through PI3 K/AKT Signaling Pathway

**Fig. 6:**
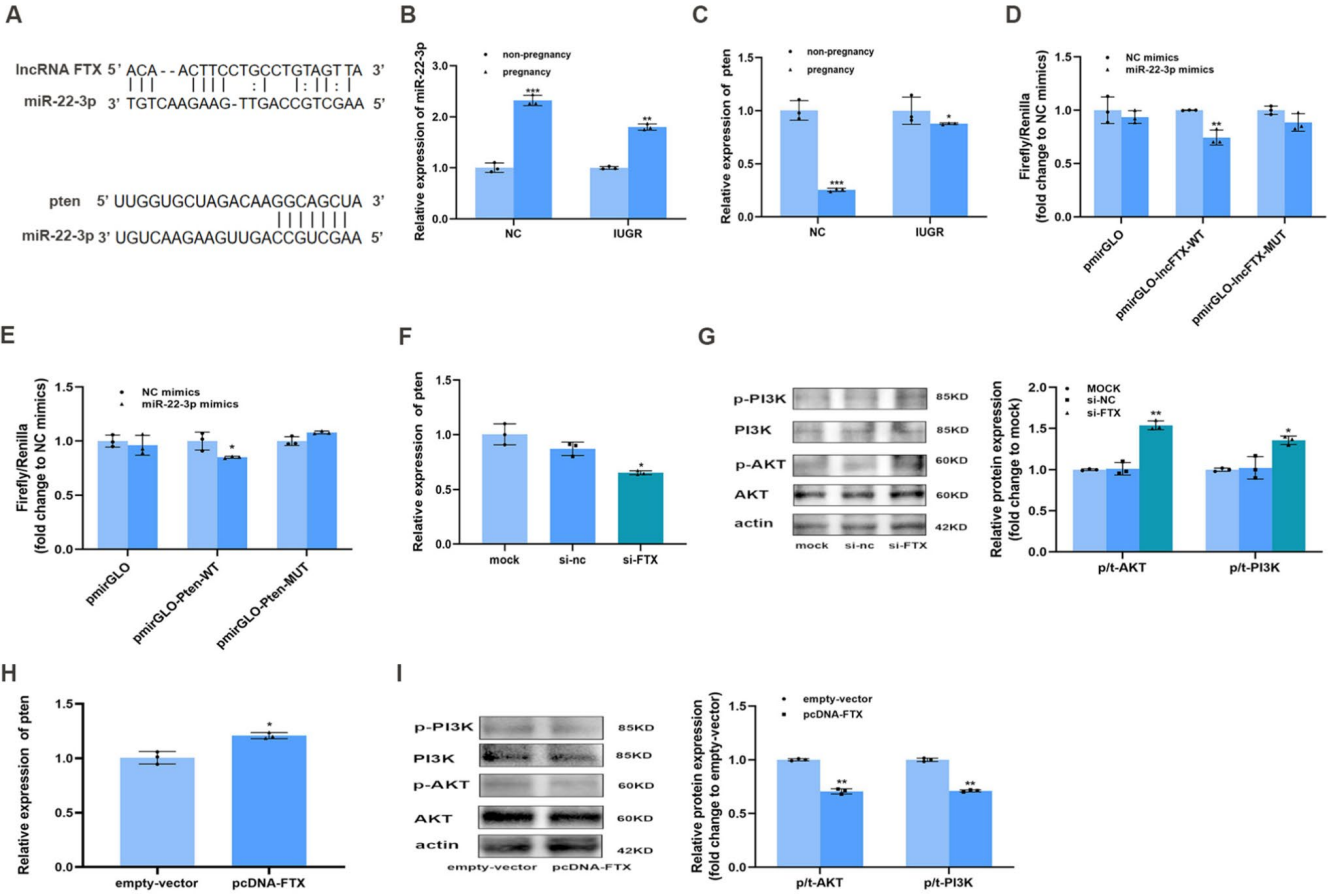
FTX was a sponge of miR- 22 - 3p which regulated pten through PI3 K/AKT signaling pathway. (**A**) StarBase V2.0 predicted that miR- 22 - 3p was a target of lncRNA FTX and pten was a target of miR- 22 - 3p. The levels of miR- 22 − 3p (**B**) and pten (**C**) in NC, NC pregnancy, IUGR, IUGR pregnancy, compared with non-pregnancy. (**D**) Dual luciferase report assay in WT- lncRNA FTX or MUT-lncRNA FTX co-transfected with miR-NC or miR- 22 - 3p mimics. (**E**) Dual luciferase assay in WT-pten or MUT-pten co-transfected with miR-NC or miR- 22 - 3p mimics, compared with NC mimics.The mRNA expression level of (**F**) pten and the protein expression levels of (**G**) p-PI3 K, PI3 K, p-AKT and AKT after down-regulation of lncRNA FTX in TC6 cells, compared with the mock group. The mRNA expression level of (**H**) pten and the protein expression levels of (**I**) p-PI3 K, PI3 K, p-AKT and AKT after the overexpression of lncRNA FTX in TC6 cells, compared with the empty-vector group. NC, Normal Control mice; IUGR, Intrauterine Growth Restriction.*p < 0.05, **p < 0.01, *p < 0.001

Previous studies have demonstrated that lncRNAs can act as gene regulators, often by functioning as microRNA sponges and influencing post-transcriptional silencing of microRNAs. Nucleocytoplasmic separation experiments confirmed that FTX was primarily expressed in the cytoplasm [13], suggesting that FTX may function in a similar regulatory manner. Bioinformatics analysis conducted by Guangzhou Gidio Biotechnology limited company suggested that lncRNA FTX interacted with Phosphatase and Tensin homolog gene (pten), a known negative regulator of the PI3 K/AKT pathway. We used StarBase V2.0 software to predict miR- 22 - 3p as a target of lncRNA FTX and pten as a target of miR- 22 - 3p (Fig. 6A). During pregnancy, the expression of MiR- 22 - 3p was up-regulated (Fig. 6B) and pten was down-regulated (Fig. 6C). TC6 cells were co-transfected by either NC mimics or miR- 22 - 3p mimics, along with luciferase reporter plasmids (pmirGLO-empty-vector, pmirGLO-lncFTX-WT, pmirGLO-lncFTX-mut, pmirGlO-pten-WT, pmirGLO-pten-mut), constructed by Shanghai Obio Biotechnology Company. After incubation for 48 h, the relative luciferase activity was measured by using the dual-luciferase reporter assay kit, and the results showed that the relative luciferase activity of FTX WT + miR- 22 - 3p mimics group (Fig. 6D) and pten WT + miR- 22 - 3p mimics (Fig. 6E) were lower, which further supported miR- 22 - 3p as a target of lncRNA FTX and pten as a target of miR- 22 - 3p. PI3 K/AKT pathway is associated with islet cell proliferation. In TC6 cells, downregulation of lncRNA FTX led to decreased pten mRNA levels (Fig. 6F) and increased protein expression of p/t-AKT and p/t-PI3 K (Fig. 6G), indicating enhanced PI3 K/AKT pathway activity. Conversely, overexpression of lncRNA FTX resulted in increased pten mRNA expression (Fig. 6H) and decreased p/t-AKT and p/t-PI3 K protein levels (Fig. 6I). These implied that lncRNA FTX may play a role in cell proliferation through the pten/PI3 K/AKT pathway.

## Discussion

The increasing incidence of diabetes has put great pressure on global economy and healthcare systems. Nevertheless, its etiology and pathogenesis remain complex and not fully understood. The development of the “Developmental Origins of Health and Disease” (DOHaD) theory has introduced a new perspective on the etiology of diabetes, emphasizing the “developmental origin of adult diseases”. Intrauterine growth restriction (IUGR) is one such condition that predisposes individuals to metabolic dysfunction. Epidemiological studies have established that individuals with a history of IUGR face a higher risk of developing insulin resistance and diabetes in adulthood [14, 15]. However, the metabolic consequences of pregnancy in these individuals remain poorly understood. During pregnancy, maternal physiological insulin resistance aims to provide sufficient glucose for the growing fetus, while compensatory responses of maternal β-cells help maintain glucose homeostasis [16]. Our study revealed that F1 IUGR pregnant mice exhibited higher blood glucose levels and lower insulin levels during pregnancy compared to normal pregnant controls. This suggests that the metabolic adaptations required for pregnancy may be compromised in individuals with a history of IUGR. Our findings not only highlight immediate disturbances in maternal glucose regulation (F1 IUGR pregnancy) but also suggest potential consequences, as such metabolic imbalances could increase the risk of diabetes in their F2 offspring.

In this study, we first established a neonatal IUGR mouse model by subjecting the mothers to a low-protein diet during pregnancy and lactation. At 12 weeks after birth, female IUGR mice (F1 generation) were mated with age-matched control males to generate an F1 IUGR pregnancy model. The normal gestational period of mice ranges from 18 to 21 days. We chose to extract the pancreas or islet cells from mice on gestational day 15, a stage corresponding to mid-to-late pregnancy. Studies have shown that in rodents, the peak proliferation of maternal pancreatic β-cells occurs on gestational day 14.5, with the expression of thousands of islet genes undergoing changes[17], including lncRNAs[9] as well as genes related to insulin synthesis and secretion [18]. During this period, pancreatic β-cells typically undergo adaptive changes, such as increased proliferation and enhanced insulin secretion. Our findings indicate that while islet proliferation occurs in both normal and F1 IUGR pregnant mice, the extent of β-cell proliferation in F1 IUGR pregnant mice was significantly lower than that observed in normal pregnant controls. These observations were accompanied by impaired glucose tolerance during pregnancy. Together, these findings suggest that F1 IUGR pregnancy is associated with impaired pancreatic adaptation and dysregulated glucose metabolism, warranting further investigation into the underlying molecular mechanisms.

Epigenetics, particularly the role of long non-coding RNAs (lncRNAs) in gene expression, has gained increasing attention. LncRNA has also been studied in pregnancy and its related complications. One study found that the expression of lncRNA TCL6 in peripheral blood of pregnant women with preeclampsia was up-regulated [19]. Meanwhile, TCL6 was up-regulated in placental tissues of pregnant women with threatened miscarriage and promoted early abortion through EGFR pathway [20]. However, clinical studies examining the effects of lncRNAs on pregnancy are often limited to placental tissues or peripheral blood, as obtaining pancreatic tissues or islets from pregnant women is extremely challenging—only possible in cases of accidental death and organ donation. To address this gap, we established an F1 IUGR pregnant mouse model and performed RNA-sequencing on islets extracted from IUGR and normal pregnant mice. Our analysis identified 1007 differentially expressed lncRNAs between the two groups. Bioinformatics analysis revealed that the target genes of these differentially expressed lncRNAs were enriched in biological pathways related to islet cell proliferation, apoptosis, and insulin secretion, suggesting a potential role in pancreatic adaptation during pregnancy. We selected highly differentially expressed lncRNA FTX to explore its role and mechanism in islet function of F1 IUGR pregnant mice.

It is well established that lncRNA expression is tissue-specific. In our study, lncRNA FTX expression in islets was significantly higher than in other tissues. To investigate whether FTX plays a regulatory role during pregnancy, we examined its expression in islets of normal and F1 IUGR mice during both non-pregnant and pregnant states. Our results revealed that FTX expression was down-regulated during pregnancy in both groups. However, the degree of down-regulation was attenuated in F1 IUGR pregnant mice compared to normal pregnant mice, which was consistent with our RNA-sequencing data.

Islet β-cells respond to abnormal blood glucose levels by altering gene expression, and it is believed that lncRNA expression is regulated by glucose concentration. Therefore, we stimulated TC6 cells with different glucose concentrations and observed that the expression level of FTX varied with changes in glucose concentration. Additionally, the transcription factors related to insulin synthesis and secretion, such as Insulin, Neurod, Glut2, and Pdx- 1, also responded to blood glucose levels. Among these, insulin is a key transcription factor for insulin synthesis. Neurod, a helix-loop-helix transcription factor, is crucial for pancreatic β-cell development and transcription of insulin gene. Neurod mutations might predispose individuals to maturity onset diabetes of the young (MODY) 6 [21]. Pdx- 1, also known as insulin promoter 1, regulates the expression of insulin-related genes such as glucose transporter 2 (glut2), promotes insulin secretion and maintains pancreatic β-cell function [22]. Our study showed that serum insulin levels increase during pregnancy, along with upregulated expression of genes associated with insulin synthesis and secretion. This suggests that the glucose-stimulated insulin secretion (GSIS) capacity of β-cells is enhanced to maintain glucose homeostasis during pregnancy. To further investigate the relationship between FTX and β-cell function, we knocked down FTX in the mouse pancreatic β-cell line TC6. This resulted in increased mRNA levels of Insulin, Neurod, Pdx- 1, and Glut2. Conversely, FTX overexpression led to decreased expression of these genes. These findings indicate that FTX influences the expression of insulin-related genes, thereby modulating the function of islet β-cells.

In our study, F1 IUGR pregnant mice exhibited a smaller insulin-staining area and higher FTX expression than normal pregnant mice, suggesting that inadequate islet proliferation may occur in IUGR pregnancy, with FTX potentially involved in this process. FTX has been implicated in tumor cell proliferation and migration, though its function varies by tissue and cancer type. Jin et al. found that FTX activated FOXA2 expression to inhibit proliferation and migration of non-small-cell lung cancer (NSCLC) [23], while Huo et al. found that FTX promoted proliferation and migration of lung adenocarcinoma cells by targeting miR- 335 - 3p/NUCB2 axis [24], yet FTX has not been studied in islet cells. We investigated whether FTX was correlated with the proliferation and apoptosis of islet cells, and observed increased cell proliferation activity, a reduced apoptosis rate, significantly decreased expression of pro-apoptotic proteins Bax and Caspase- 3, and an increase in the anti-apoptotic protein Bcl- 2 after FTX was intervened. Conversely, FTX overexpression led to decreased proliferation and increased apoptosis in TC6 cells. These results suggest that FTX might regulate islet cell proliferation and apoptosis.

To further explore the mechanism of FTX in regulating islet cell function and number, we identified that lncRNA FTX had an interaction with pten (mRNA) by bioinformatics analysis. Pten, a phosphatase tensin homolog on human chromosome 10, was originally identified as a tumor suppressor gene and is one of the most frequently mutated genes in human cancers. As a phosphoinositide phosphatase, pten dephosphorylates PtdIns (3,4,5) P3 at the inositol ring D3 to form PtdIns (4,5) P2 [25]. As a direct antagonist of PI3 K, pten is a negative regulator of the PI3 K/AKT pathway and the loss of pten drives the overactivation of PI3 K/AKT in many tumors. Pten also has protein phosphatase activity that regulates cell proliferation, cell cycle progression, apoptosis, cell adhesion, migration and invasion by interacting with target proteins [26]. Loss of pten has been widely shown to increase islet mass and promote β-cell proliferation, primarily through PI3 K/AKT pathway activation [27, 28]. The activation of PI3 K/AKT pathway increases islet β-cell mass by stimulating cell proliferation and inhibiting cell apoptosis [29], and simultaneously promotes insulin secretion of β-cells [30], alleviates insulin resistance and hepatic gluconeogenesis [31]. To verify whether FTX regulates the pten/PI3 K/AKT axis, we down-regulated lncRNA FTX in TC6 cells, and observed a decrease in pten expression, along with increased levels of p/t-AKT and p/t-PI3 K. Conversely, FTX overexpression led to increased pten expression and reduced levels of p/t-AKT and p/t-PI3 K. These suggest that lncRNA FTX regulated the function and number of islet β-cells through pten/PI3 K/AKT axis.

Bioinformatics analysis also indicated that the interaction between lncRNA and pten occurred via a “trans” mechanism, meaning that their positions in the genome are not directly related, but their functions are interconnected. The target gene of lncRNA could be predicted by co-expression analysis method or correlation analysis of their expression levels and be regulated at its transcription or post-transcription level. Competing endogenous RNAs (CeRNA) fall into this category. Some lncRNAs could bind to microRNAs as “decoys” or “sponges” to release mRNAs from the inhibition of microRNAs, thus restoring the function of mRNAs, which was vividly called “sponge” effect. Yang et al.reported that FTX reduced hypertrophy of neonatal mouse cardiac myocytes and regulated the pten/PI3 K/AKT signaling pathway by sponging MicroRNA- 22 [32]. This raised the question of whether a similar “sponge” effect occurs in islets. To investigate this, we used StarBase V2.0 software to predict miR- 22 - 3p as the target gene of lncRNA FTX and pten as the target gene of miR- 22 - 3p. Dual-luciferase reporter assays confirmed these interactions, as the relative luciferase activity was significantly reduced in both the FTX WT + miR- 22 - 3p mimic and the pten WT + miR- 22 - 3p mimic groups. These results validate miR- 22 - 3p as a target of lncRNA FTX and confirm that PTEN is indeed a target of miR- 22 - 3p.

In conclusion, our study demonstrated that islet cell proliferation and the expression of insulin-related genes increased during pregnancy in mice, though this proliferation was notably reduced in F1 IUGR pregnant mice. We observed that lncRNA FTX, which was regulated by glucose levels, was highly expressed in mouse islets and typically down-regulated during pregnancy. However, this downregulation was weaker in F1 IUGR pregnant mice than normal pregnant mice. FTX may affect the proliferation, apoptosis and function of TC6 cells through the pten/PI3 K/AKT axis after being interfered or overexpressed in TC6 cells. We predicted that there might be a “sponge” relationship between lncRNA FTX, miR- 22 - 3p, and pten, which we validated through a dual-luciferase reporter assay. This study suggests that individuals born with IUGR may have a higher risk of islet dysfunction during pregnancy. Therefore, it is recommended that clinicians closely monitor blood glucose levels in pregnant women born with IUGR to prevent the onset of gestational diabetes mellitus (GDM). Furthermore, lncRNA FTX has emerged as a promising therapeutic target for preserving or restoring pancreatic β-cell function, offering a potential RNA-based intervention for IUGR and its associated metabolic disorders, such as GDM. Notably, the identification of the FTX-miR- 22 - 3p-pten axis opens new possibilities for personalized molecular-targeted therapies. RNA-based therapeutics or small-molecule interventions targeting FTX/miR- 22 - 3p could represent an innovative strategy for improving IUGR-related islet dysfunction and metabolic outcomes.

## Conclusion

This study showed that FTX might play a regulatory role in islet function during the pregnancy of mice that were born with IUGR through the pten/PI3 K/AKT pathway.

## Data Availability

The data that support the findings of this study are available from the corresponding author upon reasonable request.

## References

[1] Hryciw DH. Early life nutrition and the development of offspring metabolic health. Int J Mol Sci. Jul 22 2022;23(15). 10.3390/ijms23158096 10.3390/ijms23158096 PMC9330842 35897668

[2] He X, Xie Z, Dong Q, Chen P, Li W, Wang T. Dynamic p53 protein expression and phosphorylation in the kidneys of rats that experienced intrauterine growth restriction. Ren Fail. 2015;37(5):896–902. 10.3109/0886022x.2015.1015428. 25721428 10.3109/0886022X.2015.1015428

[3] Boehmer BH, Limesand SW, Rozance PJ. The impact of IUGR on pancreatic islet development and β-cell function. J Endocrinol. 2017;235(2):R63-r76. 10.1530/joe-17-0076. 28808079 10.1530/JOE-17-0076 PMC5808569

[4] Golden TN, Simmons RA. Immune dysfunction in developmental programming of type 2 diabetes mellitus. Nat Rev Endocrinol. 2021;17(4):235–45. 10.1038/s41574-020-00464-z. 33526907 10.1038/s41574-020-00464-z PMC7969450

[5] Rashid CS, Bansal A, Simmons RA. Oxidative stress, intrauterine growth restriction, and developmental programming of type 2 diabetes. Physiology (Bethesda). 2018;33(5):348–59. 10.1152/physiol.00023.2018. 30109821 10.1152/physiol.00023.2018 PMC6230552

[6] Li Y, Dai C, Yuan Y, You L, Yuan Q. The mechanisms of lncRNA Tug1 in islet dysfunction in a mouse model of intrauterine growth retardation. Cell Biochem Funct. 2020;38(8):1129–38. 10.1002/cbf.3575. 32869325 10.1002/cbf.3575

[7] Plows JF, Stanley JL, Baker PN, Reynolds CM, Vickers MH. The pathophysiology of gestational diabetes mellitus. Int J Mol Sci. Oct 26 2018;19(11). 10.3390/ijms19113342. 10.3390/ijms19113342 PMC6274679 30373146

[8] Liu Y, Kuang A, Talbot O, et al. Metabolomic and genetic associations with insulin resistance in pregnancy. Diabetologia. 2020;63(9):1783–95. 10.1007/s00125-020-05198-1. 32556615 10.1007/s00125-020-05198-1 PMC7416451

[9] Sisino G, Zhou AX, Dahr N, et al. Long noncoding RNAs are dynamically regulated during β-cell mass expansion in mouse pregnancy and control β-cell proliferation in vitro. PLoS ONE. 2017;12(8):e0182371. 10.1371/journal.pone.0182371. 28796801 10.1371/journal.pone.0182371 PMC5552087

[10] Huang S, Zhu X, Ke Y, et al. LncRNA FTX inhibition restrains osteosarcoma proliferation and migration via modulating miR-320a/TXNRD1. Cancer Biol Ther. 2020;21(4):379–87. 10.1080/15384047.2019.1702405. 31920141 10.1080/15384047.2019.1702405 PMC7515494

[11] Guo B, Xu X, Chi X, Wang M. Relationship of lncRNA FTX and miR-186-5p levels with diabetic peripheral neuropathy in type 2 diabetes and its bioinformatics analysis. Ir J Med Sci. 2024;193(5):2293–9. 10.1007/s11845-024-03720-7. 38837012 10.1007/s11845-024-03720-7

[12] Huang L, Xiong S, Liu H, et al. Bioinformatics analysis of the inflammation-associated lncRNA-mRNA coexpression network in type 2 diabetes. J Renin Angiotensin Aldosterone Syst. 2023;2023:6072438. 10.1155/2023/6072438. 36874406 10.1155/2023/6072438 PMC9977555

[13] Shen Y, Yang G, Zhuo S, Zhuang H, Chen S. lncRNA FTX promotes asthma progression by sponging miR-590-5p and upregulating JAK2. Am J Transl Res. 2021;13(8):8833–46. 34539998 PMC8430149

[14] Ross MG, Beall MH. Adult sequelae of intrauterine growth restriction. Semin Perinatol. 2008;32(3):213–8. 10.1053/j.semperi.2007.11.005. 18482624 10.1053/j.semperi.2007.11.005 PMC2527863

[15] Berends LM, Dearden L, Tung YCL, Voshol P, Fernandez-Twinn DS, Ozanne SE. Programming of central and peripheral insulin resistance by low birthweight and postnatal catch-up growth in male mice. Diabetologia. 2018;61(10):2225–34. 10.1007/s00125-018-4694-z. 30043179 10.1007/s00125-018-4694-z PMC6133152

[16] Kampmann U, Knorr S, Fuglsang J, Ovesen P. Determinants of maternal insulin resistance during pregnancy: an updated overview. J Diabetes Res. 2019;2019:5320156. 10.1155/2019/5320156. 31828161 10.1155/2019/5320156 PMC6885766

[17] Rieck S, White P, Schug J, et al. The transcriptional response of the islet to pregnancy in mice. Mol Endocrinol. 2009;23(10):1702–12. 10.1210/me.2009-0144. 19574445 10.1210/me.2009-0144 PMC2754894

[18] Xue Y, Liu C, Xu Y, et al. Study on pancreatic islet adaptation and gene expression during pregnancy in rats. Endocrine. 2010;37(1):83–97. 10.1007/s12020-009-9273-0. 20963559 10.1007/s12020-009-9273-0

[19] Wang H, Shen G, Liu M, Mao L, Mao H. Expression and clinical significance of lncRNA TCL6 in serum of patients with preeclampsia. Exp Ther Med. 2022;23(1):41. 10.3892/etm.2021.10963. 34849156 10.3892/etm.2021.10963 PMC8613530

[20] Liu LP, Gong YB. LncRNA-TCL6 promotes early abortion and inhibits placenta implantation via the EGFR pathway. Eur Rev Med Pharmacol Sci. 2018;22(21):7105–12. 10.26355/eurrev_201811_16242. 30468451 10.26355/eurrev_201811_16242

[21] Gu C, Stein GH, Pan N, et al. Pancreatic beta cells require NeuroD to achieve and maintain functional maturity. Cell Metab. 2010;11(4):298–310. 10.1016/j.cmet.2010.03.006. 20374962 10.1016/j.cmet.2010.03.006 PMC2855640

[22] Kaneto H, Miyatsuka T, Kawamori D, Matsuoka TA. Pleiotropic roles of PDX-1 in the Pancreas. Rev Diabet Stud Winter. 2007;4(4):209–25. 10.1900/rds.2007.4.209. 10.1900/RDS.2007.4.209 PMC2270408 18338074

[23] Jin S, He J, Zhou Y, Wu D, Li J, Gao W. LncRNA FTX activates FOXA2 expression to inhibit non-small-cell lung cancer proliferation and metastasis. J Cell Mol Med. 2020;24(8):4839–49. 10.1111/jcmm.15163. 32176463 10.1111/jcmm.15163 PMC7176842

[24] Huo X, Wang H, Huo B, et al. FTX contributes to cell proliferation and migration in lung adenocarcinoma via targeting miR-335-5p/NUCB2 axis. Cancer Cell Int. 2020;20:89. 10.1186/s12935-020-1130-5. 32226311 10.1186/s12935-020-1130-5 PMC7092578

[25] Gericke A, Leslie NR, Lösche M, Ross AH. PtdIns(4,5)P2-mediated cell signaling: emerging principles and PTEN as a paradigm for regulatory mechanism. Adv Exp Med Biol. 2013;991:85–104. 10.1007/978-94-007-6331-9_6. 23775692 10.1007/978-94-007-6331-9_6 PMC3763917

[26] Haddadi N, Lin Y, Travis G, Simpson AM, Nassif NT, McGowan EM. PTEN/PTENP1: “Regulating the regulator of RTK-dependent PI3K/Akt signalling”, new targets for cancer therapy. Mol Cancer. 2018;17(1):37. 10.1186/s12943-018-0803-3. 29455665 10.1186/s12943-018-0803-3 PMC5817727

[27] Mziaut H, Henniger G, Ganss K, et al. MiR-132 controls pancreatic beta cell proliferation and survival through Pten/Akt/Foxo3 signaling. Mol Metab. 2020;31:150–62. 10.1016/j.molmet.2019.11.012. 31918917 10.1016/j.molmet.2019.11.012 PMC6928290

[28] Yang KT, Bayan JA, Zeng N, et al. Adult-onset deletion of Pten increases islet mass and beta cell proliferation in mice. Diabetologia. 2014;57(2):352–61. 10.1007/s00125-013-3085-8. 24162585 10.1007/s00125-013-3085-8 PMC3918745

[29] Kitagishi Y, Nakanishi A, Minami A, et al. Certain diet and lifestyle may contribute to Islet β-cells protection in type-2 diabetes via the modulation of cellular PI3K/AKT pathway. Open Biochem J. 2014;8:74–82. 10.2174/1874091x01408010074. 25400709 10.2174/1874091X01408010074 PMC4231374

[30] Liu Q, Wang R, Zhou H, et al. SHIP2 on pI3K/Akt pathway in palmitic acid stimulated islet β cell. Int J Clin Exp Med. 2015;8(3):3210–8. 26064210 PMC4443044

[31] Chen SH, Liu XN, Peng Y. MicroRNA-351 eases insulin resistance and liver gluconeogenesis via the PI3K/AKT pathway by inhibiting FLOT2 in mice of gestational diabetes mellitus. J Cell Mol Med. 2019;23(9):5895–906. 10.1111/jcmm.14079. 31287224 10.1111/jcmm.14079 PMC6714143

[32] Yang X, Tao L, Zhu J, Zhang S. Long noncoding RNA FTX reduces hypertrophy of neonatal mouse cardiac myocytes and regulates the PTEN/PI3K/Akt signaling pathway by sponging MicroRNA-22. Med Sci Monit. 2019;25:9609–17. 10.12659/msm.919654. 31840653 10.12659/MSM.919654 PMC6929539

